# Prevention and Management of Depression and Suicidal Behavior in Men with Prostate Cancer

**DOI:** 10.3389/fpubh.2015.00028

**Published:** 2015-02-19

**Authors:** Jeremy Kiffel, Leo Sher

**Affiliations:** ^1^Icahn School of Medicine at Mount Sinai, New York, NY, USA; ^2^James J. Peters Veterans’ Administration Medical Center, Bronx, NY, USA

**Keywords:** prostate cancer, cancer research, depression, suicide, psychotherapy, risk reduction behavior

## Introduction

Prostate cancer is the most common non-skin cancer in men (1). In the U.S. between 2007 and 2011, the incidence of prostate cancer was 147.8 per 100,000 men per year, and its prevalence in 2011 was estimated to be 2,707,821 (2). Additionally, 43% of the total U.S. male cancer survivor population are survivors of prostate cancer (3). Prostate cancer is diagnosed by tissue biopsy, and a patient is most often referred for a biopsy due to an abnormal screening serum prostate-specific antigen level and/or abnormal findings on digital rectal exam (4). The tumor is characterized based on its stage and grade (Gleason score). The treatment options vary based on the severity and risk of the tumor, as well as the patient’s age and co-morbid conditions, and include active surveillance, external beam radiation therapy, brachytherapy, and radical prostatectomy for early stage disease, and androgen deprivation therapy and chemotherapy for more advanced disease (5). Due to frequent early detection of the cancer, the overwhelming majority of cases (93%) involves a tumor confined to the prostate or one that has only spread to regional lymph nodes, for which the 5-year survival rate is approximately 100%. Four percent of cases of prostate cancer involve distant metastases, for which the 5-year survival rate is approximately 28% (2). Although survival rates are excellent for localized prostate cancer, there are many long-term effects of treatment that significantly impact a patient’s daily living, including urinary incontinence, sexual dysfunction, and bowel urgency (6). Additionally, patients undergoing chemotherapy for prostate cancer often experience side effects such as anemia, neutropenia, fatigue, myalgia, fever, and diarrhea, among others (7).

## Depression

Depression is known to be associated with advanced physical illnesses and high symptom burden, including cancer (8). More specifically, men who have received a diagnosis of prostate cancer and have begun treatment have a significantly increased incidence of the development of anxiety and depression compared to their counterparts who do not have prostate cancer (9, 10). The lifetime prevalence of major depressive disorder in adults in the U.S. is 17% (11), and research has shown that in patients with prostate cancer, particularly those treated with radiotherapy, the prevalence of depression is considerably higher in patients both pre-treatment (27%) and 5 years post-treatment (22%) (12). Additionally, patients with advanced cancer, as well as patients who have undergone chemotherapy, have a higher prevalence of depression (13, 14). Interestingly, there is significant variation in the psychological effects of prostate cancer depending on the age of the patient (15). Anxiety and distress have been found to be less prevalent in older patients compared to younger patients, while depression has been found to be more prevalent in the former. This indicates that young and old cancer patients react differently to the impact of having cancer and undergoing treatment (15).

## Suicide Risk

Depression is associated with an increased risk of suicide (16); about 60% of people who commit suicide suffer from depression (17). The increased incidence and prevalence of depression in men with prostate cancer is cause for concern for an increased risk of suicidal ideation and behavior in such patients. In fact, in patients diagnosed with prostate cancer, as well as those who have been treated and survived the cancer, studies have shown an increase in suicidal ideation (18). Additionally, there is an increased rate of completed suicide in cancer patients compared to the general population (19). Prostate, lung, pancreatic, and head and neck cancers have been identified as four specific malignancies that have higher suicide rates, with rates highest among older men (20). Other studies that have verified a significantly increased risk of suicide in men with prostate cancer indicate an especially increased risk within 18 months of diagnosis, even in patients with low-risk cancer, and a more prolonged risk in patients with metastases. This is true regardless of marital status, socioeconomic status, and many other risk factors (21, 22).

## Prevention and Management of Depression and Suicide

We have a number of suggestions regarding these issues. The patients and their families, as well as medical professionals, should be educated about the psychiatric risks associated with prostate cancer so the patients can be more vigilant in caring for themselves, as well as realize that what they may be experiencing is not unusual and that they can feel comfortable sharing their experiences with family and professionals who are equipped to help them. Furthermore, research has shown that educating the patient about prostate cancer, such as in details concerning diagnosis, treatment, and things he can do to improve his health (eat healthier, exercise, quit smoking), can facilitate an improved quality of life for the patient, including a reduction in depressive symptoms (23).

Additionally, the risks associated with the various treatment options should be made clear when discussing the different treatment modalities with the patient, for some are associated with more risks compared to others. For instance, hormone therapy is associated with prolonged negative effects on health-related quality of life and psychological distress compared to other treatments (24). And research has shown that patients who have undergone a major surgical operation sometimes develop subsyndromal or syndromal post-traumatic stress disorder, which is associated with depression (25). Also, 2 and 5 years following treatment, patients who have undergone radical prostatectomy are more likely to complain of urinary incontinence and erectile dysfunction, but less bowel urgency, compared to those who have undergone radiation therapy. It should be noted, however, that by 15 years after treatment those differences in urinary and sexual outcomes disappear, although they both continue to experience problems in these areas (6). Additionally, radiotherapy has been found to be associated with a relatively increased risk of depression compared to other treatments (12). Thus, these associated risks should function as part of the equation when deciding on the most appropriate treatment for a patient.

It is also important for physicians to implement techniques of management that can significantly affect their patients’ mood and behavior. One of the tools available is appropriate psychopharmacology that is focused on the treatment and management of anxiety and depression. Additionally, psychotherapy has been shown to be effective for the treatment of depression in advanced cancer patients (26). With regard to prostate cancer, cognitive behavioral stress-management interventions have been successful in improving patients’ quality of life. These interventions include anger management, identification of distorted thoughts, assertiveness training, rational thought replacement, and utilization of social support (27). Studies also show that patients who use more approach coping have better psychological and physical outcomes compared to those who use more avoidance coping. Emotion-focused coping has also been associated with better sexual function and quality of life (28).

What has become more obvious is the need to focus more on those men who most require help and support. The “broad brush” approach of offering the same interventions to well-adjusted, well-educated men who experience the same quality of life as the normal population, and to those experiencing urinary, sexual, or marital difficulties is unlikely to be effective in terms of desired outcome and cost. As survival rates improve, a large number of men will be living in the aftermath of the disease and its treatment. The challenge is to develop and test interventions aimed at helping these men.

## Conclusion

Considering how common prostate cancer is and the seriousness of its associated psychiatric risks, further research concerning the prevention and management of depression and suicidal behavior in men with prostate cancer should be conducted. There is a great need to educate the general public and medical professionals about the prevalence and gravity of these issues. Early and continual screening for symptoms of depression and suicidal behavior in patients with prostate cancer is warranted due to the significant risk and the available means to prevent and manage it.

## Conflict of Interest Statement

The authors declare that the research was conducted in the absence of any commercial or financial relationships that could be construed as a potential conflict of interest.
